# Integrated Redox Profiling: Simultaneous Determination of Ubiquinol-10, Ubiquinone-10, and Alpha-Lipoic Acid in Serum by LC-MS/MS

**DOI:** 10.3390/metabo16050344

**Published:** 2026-05-20

**Authors:** Domniki Gallou, Olga Begou, Georgios Theodoridis, Helen Gika

**Affiliations:** 1 Laboratory of Analytical Chemistry, Department of Chemistry, Aristotle University of Thessaloniki, 54124 Thessaloniki, Greece; gdomnikii@chem.auth.gr (D.G.); gtheodor@chem.auth.gr (G.T.); 2 Biomic AUTh, Center for Interdisciplinary Research and Innovation (CIRI-AUTH), Balkan Center B1.4, 10th km Thessaloniki-Thermi Rd, 57001 Thessaloniki, Greece; gkikae@auth.gr; 3 ThetaBiomarkers, Balkan Center B1.4, 10th km Thessaloniki-Thermi Rd, 57001 Thessaloniki, Greece; 4 Laboratory of Forensic Medicine & Toxicology, School of Medicine, Aristotle University of Thessaloniki, 54124 Thessaloniki, Greece

**Keywords:** method development, coenzyme Q_10_, Alpha-lipoic acid, oxidative stress

## Abstract

**Background**: Coenzyme Q_10_ and Alpha-lipoic acid are two essential antioxidants involved in numerous physiological processes, including cellular energy production and the mitigation of oxidative stress. Their accurate quantification is critical for understanding their biological roles and therapeutic potential. Herein, an RPLC-MS/MS method for the rapid and simultaneous determination of ubiquinone-10 (CoQ_10_), the reduced form ubiquinol-10 (CoQ_10_H_2_), and Alpha-lipoic acid (ALA) in human serum was developed and validated. **Methods**: Chromatographic separation was performed on a Waters ACQUITY UPLC HSS T3 column (2.1 mm × 150 mm, i.d. 1.7 μm). Detection was performed on a SCIEX Triple Quad 6500+ system, applying multiple reaction monitoring (MRM). Single-phase protein precipitation was selected as the sample preparation protocol, providing satisfactory recovery for the analytes. **Results**: The method was linear over the concentration of 53.8–613 ng/mL for CoQ_10_H_2_, 23.1–263 ng/mL for CoQ_10_ and 7.7–87.6 ng/mL for ALA. Intra- and inter-day accuracy was found to be between 81.8 and 109% and 84.4 to 106%, respectively, for all analytes, while intra- and inter-day precision was found to vary from 0.8% to 9.9% %RSD and 2.0% to 7.7% %RSD, respectively. A limit of quantitation (LOQ) of 4.2 ng/mL was found for CoQ_10_H_2_, 1.7 ng/mL for CoQ_10_ and 0.7 ng/mL for ALA. **Conclusions**: The developed LC-MS/MS method enables rapid, sensitive and simultaneous quantification of CoQ_10_H_2_, CoQ_10,_ and ALA in human serum with satisfactory accuracy, precision and sensitivity. The method is suitable for bioanalytical applications and was successfully applied to the analysis of 10 real samples obtained from healthy volunteers.

## 1. Introduction

Coenzyme Q_10_ is a naturally occurring lipid-soluble benzoquinone that plays an essential role in cellular energy metabolism. CoQ_10_ functions as a mobile electron carrier in the mitochondrial respiratory chain, transferring electrons from complex I (NADH ubiquinone reductase) and complex II (succinate ubiquinone reductase) to complex III (ubiquinol:cytochrome c reductase) during oxidative phosphorylation [1,2]. Coenzyme Q10 is synthesized endogenously via the mevalonate pathway, which is also involved in cholesterol biosynthesis, and its circulating concentrations may be influenced by dietary intake and the administration of drugs that interfere with the mevalonate pathway, such as statins [3,4], as well as pathophysiological conditions. In biological systems, Coenzyme Q10 exists mainly in two redox forms, the oxidized form, ubiquinone-10 (CoQ_10_), and the reduced form, ubiquinol-10 (CoQ_10_H_2_). Approximately 90–95% of Coenzyme Q_10_ in circulation and tissues is present in its reduced form, which functions as a potent lipid-phase antioxidant by preventing lipid preoxidation in cellular membranes and lipoproteins, including low-density lipoproteins (LDLs), through its oxidation to ubiquinone-10 [5,6]. Alterations in Coenzyme Q_10_ concentration levels, and especially in the CoQ_10_H_2_/CoQ_10_ redox ratio, have been associated with oxidative stress, mitochondrial dysfunction and several pathological conditions, including cardiovascular diseases (CVDs) [7], neurodegenerative disorders [8], diabetes type 2 [9] and metabolic syndrome [10]. For this reason Coenzyme Q_10_ has been widely studied in biological matrices, such as plasma [11,12], serum [13], tissues [14] and cell cultures [15] in both human and animal models.

A metabolite closely related to mitochondrial metabolism and antioxidant defense is alpha-lipoic acid (ALA), also known as thioctic acid. ALA is a naturally occurring disulfide compound that functions as a cofactor for several mitochondrial enzyme complexes involved in oxidative metabolism, including pyruvate dehydrogenase and a-ketoglutarate dehydrogenase [16]. ALA exhibits antioxidant properties as it can react with reactive oxygen species (ROS) and contribute to the maintenance of cellular redox balance, while supporting endogenous antioxidant systems, such as glutathione and vitamins C and E [17]. In biological systems, the majority of ALA exists in a covalently protein-bound form, typically attached to lysine residues within mitochondrial enzyme complexes, where it functions as a catalytic cofactor and only a small fraction is present as free ALA in circulation [18]. Besides its endogenous synthesis, ALA can be obtained from dietary sources and is also widely used as a nutritional supplement [16]. Following oral administration, ALA is absorbed from the gastrointestinal tract and distributed to various tissues, resulting in measurable concentrations in circulation. However, considerable inter-individual variability in circulating ALA levels has been reported after supplementation, which highlights the importance of reliable analytical methods for its determination in biological matrices [19,20,21]. Due to its properties, ALA has attracted considerable interest as a therapeutic antioxidant and is frequently investigated in relation to disorders associated with oxidative stress, including diabetes-related complications and metabolic diseases [22,23,24].

As both Coenzyme Q_10_ and ALA are involved in mitochondrial redox reactions and antioxidant defense mechanisms, their simultaneous quantification in plasma provides a comprehensive “snapshot” of the systemic redox status and mitochondrial efficiency of an organism. However, the accurate determination of these analytes in biological matrices presents several analytical challenges. Coenzyme Q_10_ is a highly hydrophobic molecule that associates with lipoproteins and cellular membranes, making its extraction from biological matrices a challenging procedure, whereas CoQ_10_H_2_ is prone to oxidation during sample handling, leading to erroneous results in the redox ratio. Similarly, ALA may undergo redox transformations and degradation, while its typically very low endogenous concentrations require highly sensitive methodologies [25]. In the literature a variety of methods exist for the determination of Coenzyme Q_10_ or ALA in biological samples. Earlier studies mainly employed High-Performance Liquid Chromatography (HPLC) coupled with ultraviolet or electrochemical detectors (HPLC-UV, HPLC-ECD), particularly for CoQ_10_, due to the electroactive properties of the quinone structure [12,26,27,28,29,30], which fall short in terms of selectivity and sample preparation simplicity. More recently, liquid chromatography tandem mass spectrometry (LC-MS/MS) methods have been developed for the determination of the two forms of CoQ_10_ in serum or plasma with sensitivities ranging from 1 ng/mL to 10 ng/mL [11,12,13,31], while others target the determination of CoQ_10_ redox status in biological tissues and cellular systems [15]. Similarly, there are few reports on ALA LC-MS determination in plasma [32,33], tissues [34], and urine [35]. Although LC-MS/MS methods have been reported for the determination of CoQ_10_ redox forms or ALA individually, these approaches generally rely on separate analytical workflows, larger sample volumes, or more complex and time-consuming sample preparation procedures, including multi-step extraction protocols, as demonstrated in Table S1. Consequently, there remains a need for a rapid, sensitive, and streamlined method capable of concurrent determination of these synergistically relevant biomarkers, using minimal volume and simplified sample handling.

Herein, we describe the development and validation of an LC-MS/MS method for the simultaneous quantification of alpha-lipoic acid together with the oxidized and reduced forms of Coenzyme Q10 in human serum. The method employs single-step protein precipitation and requires only a small volume, while providing high sensitivity and reproducibility and short analysis time. By enabling the simultaneous assessment of key mitochondrial redox and antioxidant biomarkers within a single analytical run, the proposed approach offers a practical tool for oxidative stress-related studies. The method was successfully applied in human samples, demonstrating its suitability for studies involving oxidative stress profiling.

## 2. Materials and Methods

### 2.1. Chemicals and Reagents

All reagents used in the present study were of high purity (>95%). Acetonitrile (MeCN) and methanol (MeOH) were LC-MS grade and purchased from HiPerSolv CHROMANORM^®^ (VWR chemicals, Leuven, Belgium). Isopropanol (IPA, LC-MS grade) was purchased from Thermo Fisher, Waltham, MA, USA. Ethanol (EtOH, LC-MS grade), ethyl acetate (EA) and n-hexane were supplied by Thermo Fisher, Waltham, MA, USA, Chem lab (Zedelgem, Belgium) and HiPerSolv CHROMANORM^®^ (VWR chemicals, Leuven, Belgium), respectively. Ultrapure water (H_2_O) was obtained from a Milli-Q purification system (18.2 ΜΩ cm^−1^). Formic acid (HCOOH, F.A.) and ammonium formate (HCOONH_4_ > 98–100%), used as mobile phase additives, were purchased from Sigma-Aldrich (Steinheim, Germany) and ChemLab (Zedelgem, Belgium), respectively. All analytical standards, CoQ_10_, CoQ_10_H_2_, and ALA, as well as Coenzyme Q4 and valproic acid that were used as internal standards (ISs), were purchased from Sigma-Aldrich (Steinheim, Germany).

### 2.2. Sample Collection

For the application of the method, serum samples were collected from 10 healthy individuals, during a routine serum analysis test at a collaborating laboratory. The study protocol was reviewed and approved by the Ethics Committee of the Aristotle University (Research Ethics Committee AUTH) (Protocol 115484/2026). All subjects provided written informed consent for participation in the study and the respective data collected. Dietary intake over a 48 h period and the use of supplements were recorded. All personal and sensitive data were coded and anonymized according to EU legislation. Fasting serum samples were collected from apparently healthy male and female volunteers by venipuncture of the antecubital vein into Vacutainer tubes (Becton Dickinson, Franklin Lakes, NJ, USA). Following a 12 h overnight fast, five participants received a single oral dose of 600 mg ALA, and serum samples were collected 2 h post-administration. Serum samples were also obtained from five additional volunteers who did not receive any supplementation. The study population did not include volunteers under valproic acid treatment. After collection, blood was immediately placed on wet ice and centrifuged at 5 °C and 2240× *g* for 10 min within 30 min. Serum specimens were immediately transferred to pre-labeled 1.5 mL screw-capped polypropylene tubes and stored at −80 °C until analysis, if not analyzed within 30 min.

### 2.3. Calibration Standards

A 1 mg/mL stock solution of CoQ_10_H_2_, CoQ_10,_ and Coenzyme Q4, respectively, was prepared in n-hexane, while valproic acid and ALA were prepared in methanol. From these, working solutions were generated by appropriate dilutions, with ethanol and acetonitrile at a final concentration of 0.1 mg/mL for each analyte. Subsequently, a mixture of all three analytes was prepared in ethanol, containing CoQ_10_H_2_ at 7000 ng/mL, CoQ_10_ at 3000 ng/mL and ALA at 1000 ng/mL. Successive dilutions with ethanol were performed and a series of mixture solutions were prepared at concentrations of 53.8, 80.7, 121, 182, 272, 409, and 613 ng/mL for CoQ_10_H_2_; at 23.1, 34.6, 51.9, 77.8, 117, 175, and 263 ng/mL for CoQ_10_; and at 7.7, 11.5, 17.3, 25.9, 38.9, and 58.4 ng/mL for ALA (working solutions). An internal standard mixture was prepared in ethanol at a final concentration of 500 ng/mL for Coenzyme Q4 and 50 ng/mL for valproic acid, respectively.

### 2.4. Quality Control (QC) Samples

A pooled serum sample was prepared from ten different healthy individuals. The sample was used to prepare a series of spiked quality control samples (QCs). QC series were prepared at three concentration levels: low-quality control (LQC), medium-quality control (MQC) and high-quality control (HQC) by spiking 50 μL of the pooled serum sample with 50 μL of the appropriate working solution mixture. The sample was spiked at 53.8 ng/mL (LQC), 182 ng/mL (MQC) and 613 ng/mL (HQC) for CoQ_10_H_2_; at 23.1 ng/mL (LQC), 77.8 ng/mL (MQC) and 263 ng/mL (HQC) for CoQ_10_; and at 7.7 ng/mL (LQC), 25.9 ng/mL (MQC) and 87.6 ng/mL (HQC) for ALA.

### 2.5. Analytical Conditions and Instrumentation

Reverse-Phase Liquid Chromatography tandem Mass Spectrometry (RP-LC MS/MS) was used for the quantitative analysis of the analytes. Chromatography was performed on an ACQUITY ultra performance liquid chromatography system (Waters, Milford, MA, USA) equipped with a Waters ACQUITY UPLC HSS T3 column (2.1 mm × 150 mm, 1.7 μm, Waters Corp., (Milford, MA, USA) protected by an ACQUITY UPLC VanGuard pre-column (Waters Ltd., Elstree, UK). The mobile phase consisted of a binary solvent system, where solvent A was H_2_O with 10 mM HCOONH_4_ (pH 5) and solvent B was MeCN:IPA, 1:1 (*v*/*v*), with 0.1% HCOOH. A linear gradient elution step was applied from 20 to 100% B (0–1 min) followed by an isocratic step at 100%B (1.5–6.5 min) and a 4 min equilibration step. Flow rate, column temperature and injection volume were set at 0.4 mL/min, 55 °C and 5 μL, respectively. The autosampler’s temperature was set at 10 °C. Syringe and valve washes were performed before and after every injection cycle with a strong organic solvent consisting of MeCN:IPA, 1:1 (*v*/*v*). The system was coupled to a SCIEX Triple Quad 6500+ system (AB Sciex, Framingham, MA, USA), operated in positive (ESI+) and negative (ESI−) electrospray ionization mode under OS Sciex software (version 3.1.6, Sciex, Framingham, MA, USA). Multiple reaction monitoring (MRM) mode was applied for the detection of all analytes by monitoring the transitions of *m*/*z* 883 to *m*/*z* 197 for CoQ_10_H_2_ (ESI+), *m*/*z* 881 to *m*/*z* 197 for CoQ_10_ (ESI+), *m*/*z* 205 to *m*/*z* 171 for ALA (ESI−), *m*/*z* 455 to *m*/*z* 197 for Coenzyme Q4 (ESI+), and *m*/*z* 143 to *m*/*z* 143 for valproic acid (ESI−). The source parameters were manually optimized. Curtain gas was set at 20.0 psi, collision gas was set at 5.0 psi, ion source gas 1 and 2 at 20.0 psi and 15.0 psi, respectively, source temperature at 350 °C and ion spray voltage at +4.5 kV for positive and −4.0 kV for negative ionization mode (ESI−). Declustering potential (DP) and collision energy (CE) were optimized for every single analyte and IS separately, by direct infusion of low-concentration standards into the mass spectrometer.

### 2.6. Sample Preparation

To account for the polarity gradient between the polar ALA and lipophilic CoQ_10_, two extraction strategies were evaluated: simple protein precipitation using isopropanol and liquid–liquid extraction (LLE) using either n-hexane or ethyl acetate, as described below. The applied protocols were evaluated in terms of recovery at three different concentration levels (LQC, MQC, HQC), clean-up efficiency and stability of the analytes, ensuring optimal analytical reliability and sensitivity. All sample preparation steps were performed on ice and protected from light to prevent oxidative degradation.

#### 2.6.1. Protein Precipitation

Samples were left to thaw on wet ice. A total of 20 μL of internal standard and 180 μL of isopropanol were added into 50 μL of pooled serum and the mixture was vortexed for 2 min. The mixture was then centrifuged at 6720*× g* for 4 min at 4 °C and thereafter, 175 μL of the supernatant was collected and 25 μL of ethanol was added. The final extract was transferred into an amber autosampler glass vial with a 200 μL microinsert and was subjected to LC-MS/MS analysis.

#### 2.6.2. Liquid–Liquid Extraction (LLE)

LLE was also applied for the extraction of the two forms of Coenzyme Q_10_ and ALA from serum matrix. Briefly, 50 μL of pooled serum was mixed with 20 μL of internal standard and 180 μL of isopropanol, followed by the addition of 1500 μL of either n-hexane or ethyl acetate. The mixture was vortexed for 2 min and centrifuged at 6720*× g* for 4 min at 4 °C. After centrifugation, 1000 μL of the supernatant were collected and evaporated to dryness. The dry residue was reconstituted with 50 μL of ethanol and transferred to amber LC-MS vials for analysis.

### 2.7. Recovery and Matrix Effect Evaluation

Extraction recovery and matrix effect were evaluated for the three analytes. For the assessment of extraction recovery, the pooled serum sample was spiked with analytes’ standard mixture before and after the extraction/protein precipitation step at three concentration levels (LQC, MQC, HQC). Percentage recovery, RE (%), was calculated based on the equation that is described below (Equation (1)). Matrix effect (ME(%)) was also assessed at three different concentration levels (LQC, MQC, HQC) by the analysis (*n* = 3) of the pooled serum sample, the pooled sample spiked after the extraction/protein precipitation step and the neat standard solutions at the same concentration levels. Calculation of matrix effect, ME (%), was based on Equation (2). Recovery and matrix effect were considered acceptable when values ranged between 80% and 120%.

(1) $$RE\;(\%)=\frac{\mathrm{Analyte}\;\mathrm{peak}\;\mathrm{area}\;\mathrm{spiked}\;\mathrm{before}\;\mathrm{extraction}}{\mathrm{Analyte}\;\mathrm{peak}\;\mathrm{area}\;\mathrm{spiked}\;\mathrm{after}\;\mathrm{extraction}}\times 100$$

Equation (1): Formula used for the calculation of extraction recovery.

(2) $$ME\;\left(\%\right)=\frac{\mathrm{Analyte}\;\mathrm{peak}\;\mathrm{area}\;\mathrm{spiked}\;\mathrm{after}\;\mathrm{extraction}-\mathrm{Analyte}\;\mathrm{peak}\;\mathrm{area}\;\mathrm{of}\;\mathrm{the}\;\mathrm{pooled}\;\mathrm{unspiked}\;\mathrm{sample}}{\mathrm{Analyte}\;\mathrm{peak}\;\mathrm{area}\;\mathrm{of}\;\mathrm{standard}\;\mathrm{solution}}\times 100$$

Equation (2): Formula used for the calculation of matrix effect.

### 2.8. Method Validation

Method validation experiments were performed based on the European Medicines Agency (EMA) [36], Food and Drug Administration (FDA) [37] and the International Council for Harmonisation of Technical Requirements for Pharmaceuticals for Human Use (ICH) [38] guidelines on bioanalytical method validation. Calibration, working range, limit of detection (LOD) and limit of quantification (LOQ) were established. A pooled serum sample (QC) spiked at LQC, MQC and HQC was used to evaluate precision, accuracy, and stability.

#### 2.8.1. Calibration, LOD, and LOQ

Calibration by standard addition and by external calibration curve was applied and the accuracy of determination was assessed. The concentration ranges were selected based on the reported literature values [39]. The calibration model was constructed based on the peak area ratios of the analytes to the corresponding IS (Coenzyme Q4 was used for Coenzyme Q_10_ and valproic acid for ALA) at seven concentration levels, covering the expected endogenous concentrations (Section 2.3). LOQs were established at the lower levels, where peaks were well defined and accuracy and precision of determination were within the acceptable levels (≤±20%).

#### 2.8.2. Accuracy and Precision 

For the experimental calculation of accuracy and precision, spiked QC serum samples with analyte mixtures at three different concentration levels (LQC, MQC, HQC) were analyzed. Intra-day precision and accuracy were estimated within the same day at three different concentration levels (*n* = 3), while the inter-day experiment took place over a period of three consecutive days (*n* = 3). Accuracy was calculated as the percentage ratio of the mean experimental value to the nominal value (R (%)), while precision was expressed as % relative standard deviation (%RSD) of the measurements. Accuracy was considered acceptable when the calculated concentration was within 15% of the nominal values for the QC sample, while precision was acceptable if it did not exceed 15% for every QC sample. At the LQC level, a 20% tolerance threshold for both was adopted.

#### 2.8.3. Stability 

Short-term stability of the analytes in the autosampler was assessed at three different concentration levels (LQC, MQC, HQC). The stability was evaluated for extracted spiked serum samples kept in the autosampler for 4 h and 8 h at 10 °C. The measured concentrations were compared with freshly prepared samples, and stability was expressed as % accuracy. Analyte stability was confirmed when measured concentrations were within ±15% of the nominal concentrations, while a ±20% acceptance limit was applied for the low-quality control (LQC) samples.

## 3. Results

### 3.1. LC-MS/MS Method Development

**Figure 1:**
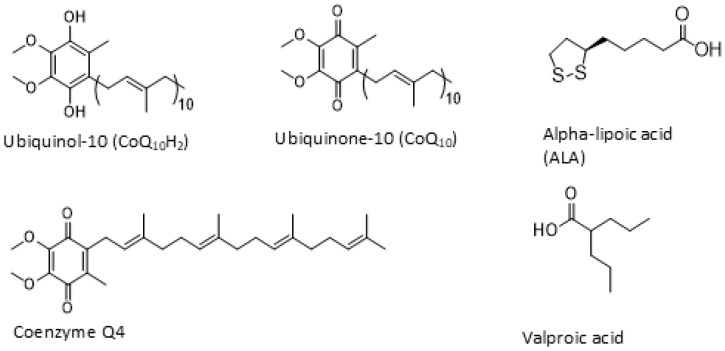
Molecular structures of the three analytes, namely Ubiquinol-10, Ubiquinone-10, Alpha-lipoic acid, and their respective internal standards, Coenzyme Q4 and Valproic acid.

Several chromatographic conditions were tested to optimize the separation and detection of the three analytes. The mobile phase of various compositions was evaluated, including aqueous ammonium formate buffer (10 mM, pH 5), combined with organic mixtures consisting of MeOH/IPA (1:1 *v*/*v*) or MeCN/IPA (1:1 *v*/*v*) + 0.1% formic acid. Three reverse-phase columns of the same dimensions were evaluated, namelyAcquity BEH C18 (1.8 μm, 2.1 × 100 mm), CSH Phenyl Hexyl (1.8 μm, 2.1 × 100 mm) and HSS T3 (1.8 μm, 2.1 × 100 mm), under different elution conditions. From all the evaluated conditions, the Acquity HSS T3 column provided superior chromatographic separation, with good peak shapes under the selected elution conditions for all three analytes, whose molecular structures are presented in Figure 1. The maximum retention time (t_R_) difference between the two forms was 0.1 min with the CSH Phenyl Hexyl column, whereas the HSS T3 column achieved a separation of approximately 0.4 min.

The optimum chromatographic separation was achieved using the binary mobile phase system consisting of A:H_2_O + 10 mM ammonium formate and B:MeCN: IPA (1:1 *v*/*v*) + 0.1% formic acid, delivered at a flow rate of 0.4 mL/min. The selected chromatographic conditions provided improved ionization efficiency and reproducible retention times of well-separated CoQ_10_H_2_ and CoQ_10_ within a short analytical run of 6.5 min in total.

Detection parameters were selected by direct infusion of freshly prepared standard solutions of the analytes into the mass spectrometer. Coenzyme Q4 was chosen as the internal standard for Coenzyme Q_10_ due to their structural similarity. Before the analysis, the absence of Coenzyme Q4 in human serum samples was verified, and no detectable levels of Coenzyme Q4 were confirmed. Detection of CoQ_10_H_2_ and CoQ_10_ was based on the formation of a positively charged adduct with ammonium formate [M+NH_4_]^+^ in positive electrospray ionization mode (ESI+). The product ion spectrum for both CoQ_10_ and CoQ_10_H_2_ provided *m*/*z* 197 as the most abundant ion, representing a benzylium ion derived from the loss of the lipophilic isoprenyl sidechain. Coenzyme Q4 was detected as a positively charged precursor ion [M+H]^+^ in positive electrospray ionization mode (ESI+), also displaying *m*/*z* 197 as the most abundant product ion. Valproic acid was selected as an internal standard for ALA due to their shared carboxylic acid functionality and similar pKa values ensuring comparable ionization efficiency and retention behavior. While it is a prescribed anticonvulsant, it is not an over-the-counter medication and is typically restricted to specific clinical populations. However, confirming its absence in the studied samples is required. Detection of ALA and valproic acid was based on the negatively charged precursor ion [M-H]^−^ in negative electrospray ionization (ESI−) mode. The product ion scan for ALA revealed the clear and abundant ion *m*/*z* 171, while the product ion scan for valproic acid (*m*/*z* 143) produced only low-abundance ions *m*/*z* 71 and *m*/*z* 80. Thus, a pseudo-MRM approach was selected for valproic acid by monitoring the transition of *m*/*z* 143 to *m*/*z* 143. For the analytes, the second most abundant ion was also monitored for confirmation purposes (qualitative ion). MRM transitions, respective chromatographic retention times and optimized MS parameters for all analytes and their respective IS are presented in Table 1.

Analysis of blank solutions showed no interferences in the respective MRM channels from the co-analytes or ISs. Retention time was calculated at 4.63 ± 0.02 min (0.32% RSD) for CoQ_10_H_2_, 4.98 ± 0.02 min (0.35% RSD) for CoQ_10_ and 1.96 ± 0.01 min (0.55% RSD) for ALA. Retention time for the internal standards was 2.85 ± 0.01 min (0.34% RSD) for Coenzyme Q4 and 2.13 ± 0.01 min (0.49% RSD) for valproic acid. The %RSD values were in all cases below 0.6%.

**Figure 2:**
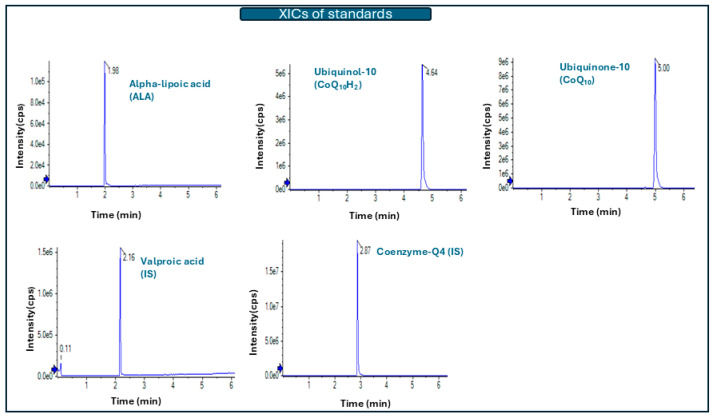
Extracted Ion Chromatograms (XICs) of a standard solution in neat solvent (38.92 ng/mL for Apha-lipoic acid, 272 ng/mL for Ubiquinol-10, 117 ng/mL for Ubiquinone-10, 50 ng/mL for Valproic acid (IS) and 500 ng/mL for Coenzyme Q4 (IS).

With regard to carryover effect, when ethanol was injected after an HQC extracted serum sample, no peaks were observed. An Extracted Ion Chromatogram (XIC) for all analytes and ISs is illustrated in Figure 2**,** where 8.92 ng/mL of ALA, 272 ng/mL of CoQ_10_H_2_ and 117 ng/mL of CoQ_10_ were prepared in neat solvent (ethanol). Valproic acid (IS) was used at 50 ng/mL and Coenzyme Q4 (IS) at 500 ng/mL.

### 3.2. Sample Preparation Optimization

During sample preparation method development, key considerations included the prevention of oxidation of the target analytes and achievement of efficient extraction, while minimizing matrix-related interferences and protecting the analytical system. A critical challenge in CoQ_10_ analysis is the rapid oxidation of CoQ_10_H_2_ into CoQ_10_; consequently, stringent measures are required to preserve the endogenous redox status of the sample.

Here, the simultaneous determination of the analytes had an additional challenge due to their disparate physicochemical properties. To address these challenges, two strategies were followed: one with a simple protein precipitation step with isopropanol and one with LLE using either n-hexane or ethyl acetate following the previously published methods [11,13,30].

**Figure 3:**
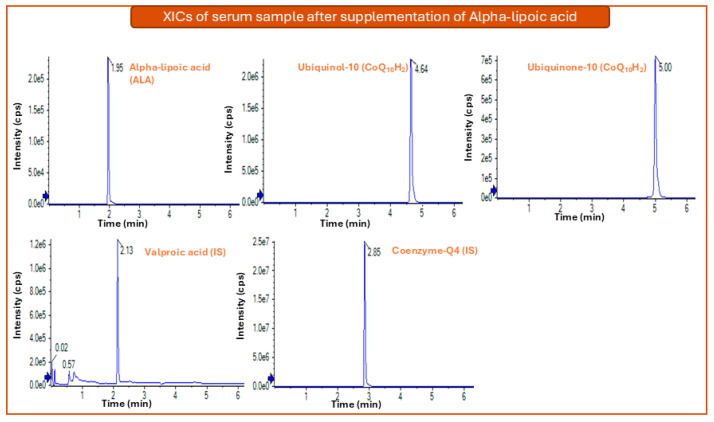
Extracted Ion Chromatograms (XICs) of a real serum sample after oral supplementation of Alpha-lipoic acid, spiked only with the internal standards (ISs).

Initial evaluations revealed that LLE with n-hexane yielded inadequate recoveries, particularly for ALA, due to its limited partitioning into the non-polar solvent. Conversely, while ethyl acetate provided satisfactory recovery for ALA, it proved inefficient for the CoQ_10_ redox pair. Notably, both LLE protocols necessitated an evaporation step, which likely compromised the stability of CoQ_10_H_2_ and CoQ_10_. These findings align with the existing literature, which frequently cites sub-optimal recoveries and analyte degradation when solvent evaporation is employed [11]. Protein precipitation using isopropanol on the other hand achieved optimal recovery for all analytes. This one-step method maintained analyte solubility, while minimizing sample handling, which is critical for preventing the oxidation of CoQ_10_H_2_. Thus, the latter procedure was adapted, as described in Section 2.6.1. In Figure 3 the chromatogram of a serum sample after oral supplementation of ALA is illustrated.

### 3.3. Method Validation 

#### 3.3.1. Calibration, Linearity, LOD, and LOQ

For calibration, two curves were applied, one external and one with the standard addition method using a pooled serum sample, at seven concentration levels, which were adapted for each analyte according to the expected concentrations in the serum samples. In Table 2, results from regression analysis for all analytes, along with the coefficients of determination (R^2^), LODs and LOQs, are presented. R^2^ values were found to be 0.997 for CoQ_10_H_2_ and CoQ_10_ and 0.999 for ALA. LOD was determined at 1.2 ng/mL for CoQ_10_H_2_, at 0.5 ng/mL for CoQ_10_ and at 0.2 ng/mL for ALA, while LOQ was determined at 4.2 ng/mL for CoQ_10_H_2_, 1.7 ng/mL for CoQ_10_ and 0.7 ng/mL for ALA. Calibration curves for all analytes in spiked pool samples are illustrated in Figure S1.

#### 3.3.2. Recovery and Matrix Effect

Matrix effect and percentage recovery for the applied protocol were evaluated. Highly satisfactory recovery was obtained for all three analytes ranging from 88.4 to 107% with the simple protein precipitation step. Noticeable matrix effects were present, particularly for CoQ_10_H_2_, where ion suppression resulted in responses of 54.1%, 67.6% and 74.9% for LQC, MQC, and HQC, respectively. However, the analytical procedure was deemed appropriate as the method sensitivity met the requirements for serum analysis and the internal standard successfully accounted for matrix interference. Table 3 presents the results from extraction recovery (RE%) and matrix effect (ME%) assessment at three concentration levels in spiked serum samples.

#### 3.3.3. Accuracy and Precision 

The developed method was assessed based on intra-day and inter-day accuracy and precision over three consecutive days. Acceptable intra- and inter-day accuracy and precision values were demonstrated through the study. Values for intra- and inter-day accuracy were found to be between 81.8 and 109% and 84.4 and 106%, respectively, for all analytes, while intra- and inter-day precision was found to vary from 0.8% to 9.9% %RSD and 2.0% to 7.7% %RSD, respectively. Table 4 summarizes the respective intra-day and inter-day accuracy and precision data.

#### 3.3.4. Stability 

Stability of the analytes in pooled extracted serum samples was also evaluated at three different concentration levels. Samples were prepared and analyzed following collection without being subjected to long-term storage or multiple freeze–thaw cycles; thus, stability assessment focused only on short-term stability to mirror the actual analytical workflow. Precautions were taken throughout sample handling and preparation to minimize oxidation of redox-sensitive analytes, particularly CoQ_10_H_2_. Serum samples were rapidly processed, transported on wet ice and analyzed promptly following collection.

Stability was assessed in the autosampler at 10 °C over a period of 4 h and 8 h. Stability was expressed as mean accuracy and found to be between 84.8 and 90.8% for CoQ_10_H_2_, 98.5 and 115% for CoQ_10_, and 102 and 110% for ALA after 4 h, and between 82.7 and 87.0% for CoQ_10_H_2_, 115 and 118% for CoQ_10_, and 102 and 107% for ALA after 8 h. The results for all QC levels for short-term stability at both time points are provided in Table 5.

### 3.4. Proof-of-Concept Application

The developed method was applied in serum samples from healthy individuals that included two groups, a supplemented group (group 1, *N* = 5) receiving ALA and a non-supplemented group (group 2, *N* = 5). For proof of concept, blood samples were collected 2 h after oral administration of 600 mg ALA. Quantifications were performed using a standard addition calibration curve.

ALA was not detected in any of the samples of the non-supplemented group. In contrast, in the supplemented group, serum concentrations ranged from 350 to 7700 ng/mL. From these, one sample exhibited a markedly higher concentration of 7700 ng/mL, suggesting inter-individual variability in absorption kinetics and bioavailability of the supplement, as previous studies have reported [20]. CoQ_10_ concentrations ranged from 35.1 to 52.7 ng/mL (0.04–0.06 μM) and CoQ_10_H_2_ from 599 to 1000 ng/mL (0.7–1.2 μΜ) in the supplemented group. The respective concentrations in the non-supplemented group were from 23.7 to 58.8 ng/mL (0.030.07μΜ) for CoQ_10_ and from 383 to 1504 ng/mL (0.4–1.7 μΜ) for CoQ_10_H_2_. Regarding CoQ_10_H_2_/CoQ_10_ ratio, it was found to vary from 14.2 to 22.1 in group 1 and from 14.4 to 37.3 in group 2, whereas the sum of the two redox forms of Coenzyme Q_10_, expressed as Total Q_10_, was found to be between 634 and 1050 ng/mL in group 1 and between 409 and 1560 ng/mL in group 2. Table 6 summarizes the results of the proof-of-concept application.

## 4. Discussion

The present study describes the development and validation of a rapid and sensitive LC-MS/MS method for the simultaneous quantification of ubiquinol-10, ubiquinone-10, and alpha-lipoic acid in human serum. Simultaneous determination of these analytes is analytically challenging because of their markedly different physicochemical properties and the instability of the reduced CoQ_10_ form during sample handling. The proposed method addresses these limitations through a simple analytical workflow based on a single-step protein precipitation protocol, enabling reliable assessment of mitochondrial redox-related biomarkers in a single analytical run.

Chromatographic conditions were optimized to accommodate the marked lipophilicity of CoQ_10_H_2_ and CoQ_10_ alongside the more polar ALA. In alignment with established methods [11,13], a reversed-phase system was selected. Although C18 columns are conventionally preferred for CoQ_10_ analysis due to their high carbon loading, C8 phases have also been used [40]. In most of the published methods, ubiquinones are eluted under isocratic gradient, with mobile phases primarily composed of lower alcohols, usually with the addition of formic acid to promote protonation in positive ionization mode or/and ammonium formate, which leads to the formation of high-abundance ammonium adducts of ubiquinones [11,13]. Similarly, previously reported methods for ALA analysis mainly employ C18 stationary phases and acidic mobile phase modifiers to ensure adequate ionization efficiency [33,34].

Although ALA exhibits a lipophilic character due to the five-carbon chain, the presence of the ionizable carboxyl group renders it relatively polar, when compared to highly lipophilic molecules of CoQ_10_H_2_ and CoQ_10_. Achieving adequate retention for ALA, while ensuring the elution of the strongly retained Coenzyme Q_10_ within a reasonable timeframe, requires a carefully optimized gradient elution and precise control of the mobile phase pH. Among the evaluated stationary phases, the HSS T3 column provided enhanced retention of the polar ALA, while maintaining strong hydrophobic interactions, making it suitable for the two forms (reduced and oxidized form) of Coenzyme Q_10_ analytes. On the other hand, BEH C18 did not provide adequate retention for ALA, and CSH Phenyl Hexyl did not show enhanced separation of the two forms of CoQ_10_ as it was expected due to the additional interactions with the aromatic rings. The use of ammonium formate in the aqueous phase further promoted stable ammonium adduct formation for ubiquinones, enhancing sensitivity and signal reproducibility.

In parallel with chromatographic optimization, sample preparation conditions were carefully evaluated to minimize analyte degradation and ensure reliable simultaneous quantification. Prevention of target analyte oxidation was critical due to the susceptibility of CoQ_10_H_2_ to oxidation, while simultaneous determination of the analytes was challenging because of their disparate physicochemical properties. For the extraction of Coenzyme Q10 from human plasma or serum, liquid–liquid extraction with n-hexane or ethyl acetate has been used, while there are reports on solid-phase extraction, which may pose increased risk of oxidation. Simpler protocols involve protein precipitation with solvents, such as 1-propanol or isopropanol. Similarly, for ALA, liquid–liquid extraction with ethyl acetate, diethyl ether, dichloromethane and tert-butyl methyl ether has been used, while protein precipitation is performed with methanol or acetonitrile [41]. In the present study, LLE with either n-hexane or ethyl acetate resulted in poor recovery for ALA and the CoQ_10_ redox pair, respectively, while the extra step of evaporation increased the risk of CoQ_10_H_2_ oxidation. In contrast, the quick, single-step protein precipitation protocol minimized sample handling and provided satisfactory recovery and reproducibility for all analytes. Recovery values ranged from 87.1% to 107% across the tested concentration levels demonstrated the suitability of the simplified extraction procedure for the simultaneous analysis.

The proof-of-concept application demonstrated the practical applicability of the method for human serum analysis and oxidative stress-related studies. The concentrations measured in the present study are in line with the results of previous studies, for alpha-lipoic acid after oral supplementation, as well as for CoQ_10_H_2_ and CoQ_10_ [11,13]. The results showed the predominance of CoQ_10_H_2_ in both groups. Even within the non-supplemented group, CoQ_10_H_2_ accounts for approximately 94% to 96% of the total CoQ_10_ plasma pool. This high ratio reflects the physiological “redox state” characteristic of a healthy organism, as the body prioritizes the reduced form due to systemic antioxidant capacity maintenance. It is noteworthy that the oxidized form CoQ_10_ remains at consistently low levels in both cases. This stability is attributed to homeostatic mechanisms for cellular protection against oxidative stress.

## 5. Conclusions

This study presents a validated RPLC-MS/MS assay that streamlines the analysis of ALA and Coenzyme Q_10_ metabolites. The combination of minimal sample requirements, low detection limits, and a simplified preparation protocol makes this method highly suitable for routine bioanalytical applications. Ultimately, this approach provides a robust analytical framework for investigating the synergistic roles of these analytes in systemic oxidative stress nutritional supplementation and metabolic disorders.

## Figures and Tables

**Table 1:** MS detection parameters and retention time (t_R_) for the analytes and their internal standards (ISs).

Analyte	t_R_ (min)	ESI Mode	MW	Precursor Ion (*m*/*z*)	Product Ion (*m*/*z*)	DP (volts)	EP (volts)	CE (eV)	CXP (volts)
Ubiquinol-10	4.63	ESI+	865.34	883	197 *	20	10	28	17
					81	20	10	28	17
Ubiquinone-10	4.98	ESI+	863.34	881	197 *	20	10	26	11
					81	20	10	26	11
Alpha-lipoic acid	1.96	ESI−	206.33	205	171 *	−25	−10	−14	−15
					64	−25	−10	−14	−15
Coenzyme Q4	2.85	ESI+	454.60	455	197 *	20	10	23	10
Valproic acid	2.13	ESI−	144.21	143	143	−25	−10	−5	−8

* Quantifier ion; t_R_: Retention Time; ESI: Electrospray Ionization; DP: Declustering Potential; EP: Exit Potential; CXP: Collision Exit Potential.

**Table 2:** Linear range (ng/mL), linear equations, coefficient of determination (R^2^) for the two constructed calibration curves, LODs (ng/mL) and LOQs (ng/mL) for all analytes.

Analyte	Calibration Range (ng/mL)	Calibration Curve	Linear Equation	R^2^	LOQ (ng/mL)	LOD (ng/mL)
Ubiquinol-10	53.8–613	external	y = 0.0027x + 0.0335	0.997	4.2	1.2
		serum	y = 0.0008x + 0.0013	0.996		
Ubiquinone-10	23.1–263	external	y = 0.0062x + 0.0335	0.997	1.7	0.5
		serum	y = 0.0034x + 0.0108	0.996		
Alpha-lipoic acid	7.7–87.6	external	y = 0.0022x + 0.0011	0.999	0.7	0.2
		serum	y = 0.0022x + 0.0008	0.999		

**Table 3:** Mean extraction recovery (RE%) and matrix effect (ME%) at three concentrations for all analytes in the spiked pool human serum sample with the applied sample preparation protocol using single-phase extraction through protein precipitation.

Analyte		Nom. Conc.(ng/mL)	RE% ± SD	ME% ± SD
Ubiquinol-10	LQC	53.8	100 ± 6	54.1 ± 9.3
	MQC	182	107 ± 8	67.6 ± 4.3
	HQC	613	105 ± 1	74.9 ± 2.4
Ubiquinone-10	LQC	23.1	96.5 ± 0.5	85.0 ± 2.2
	MQC	77.8	88.4 ± 0.8	86.3 ± 1.0
	HQC	263	87.1 ± 4.3	86.9 ± 1.7
Alpha-lipoic acid	LQC	7.7	104 ± 1	115 ± 1
	MQC	25.9	94.4 ± 0.8	108 ± 8
	HQC	87.6	106 ± 2	114 ± 2

**Table 4:** Mean concentration, accuracy, and intra- and inter-day precision (*n* = 3 × 3) for all analytes at three concentration levels.

			Day 1			Day 2			Day 3			Inter-Day		
Analyte		Nominal Conc. (ng/mL)	Mean conc. ± SD (ng/mL)	%RSD	Accuracy (RE (%))	Mean conc. ± SD (ng/mL)	%RSD	Accuracy (RE (%))	Mean conc. ± SD (ng/mL)	%RSD	Accuracy (RE (%))	Mean conc. ± SD (ng/mL)	%RSD	Accuracy (RE (%))
Ubiquinol-10	LQC	53.8	44.0 ± 0.9	2.0	81.8	45.3 ± 2.1	4.6	84.2	47.0 ± 1.6	3.4	87.3	45.4 ± 1.9	4.4	84.4
	MQC	182	176 ± 17	9.9	97.0	156 ± 2	0.9	86.0	163 ± 6	3.8	89.8	165 ± 13	7.7	90.9
	HQC	613	587 ± 20	3.4	95.8	593 ± 38	6.3	96.8	629 ± 11	1.7	103	603 ± 29	4.7	98.4
Ubiquinone-10	LQC	23.1	20.3 ± 1	5.6	87.9	19.0 ± 0.4	2.4	82.4	20.4 ± 1.0	4.8	88.6	19.9 ± 1.0	5.2	86.3
	MQC	77.8	83.1 ± 1	1.4	107	84.4 ± 4	5.0	109	78.8 ± 3.0	3.4	101	82.1 ± 4	4.4	106
	HQC	263	248 ± 8	3.1	94.5	269 ± 6	2.2	102	253 ± 8	3.3	96.2	257 ± 12	4.4	97.7
Alpha-lipoic acid	LQC	7.7	7.5 ± 0.4	5.3	97.0	8.4 ± 0.10	0.8	109	8.1 ± 3.6	3.5	105	8.0 ± 0.5	5.8	104
	MQC	25.9	27.4 ± 0.8	3.1	106	25.7 ± 0.3	1.0	99.1	25.8 ± 0.4	1.4	99.6	26.3 ± 0.9	3.6	101
	HQC	87.6	86.6 ± 0.9	1.0	98.9	88.2 ± 2.6	2.9	101	87.2 ± 1.5	1.8	99.6	87.6 ± 1.7	2.0	100

**Table 5:** Stability study of the analytes at 4 and 8 h in the autosampler (10 °C).

		4 h in 10 °C		8 h in 10 °C	
Compound		%RE	%RSD	%RE	%RSD
Ubiquinol-10	LQC	84.8	7.1	82.7	2.5
	MQC	87.6	4.1	87.0	1.0
	HQC	90.8	5.4	86.6	0.5
Ubiquinone-10	LQC	99.2	3.5	118	2.7
	MQC	98.5	2.3	108	9.4
	HQC	115	0.9	115	1.3
Alpha-lipoic acid	LQC	109	7.3	102	2.7
	MQC	112	3.9	107	2.8
	HQC	110	3.6	105	2.8

**Table 6:** Serum redox distribution of Coenzyme Q10. Concentrations of alpha-lipoic acid, ubiquinol-10, and ubiquinone-10; ubiquinol-10/ubiquinone-10 and Total Q10 measured in the samples. Concentration values are expressed as ng/mL. Samples are categorized into two groups (group 1 and group 2).

Sample	Group	Alpha-Lipoic Acid (ng/mL)	Ubiquinol-10 (ng/mL)	Ubiquinone-10 (ng/mL)	Ubiquinol-10/ Ubiquinone-10	Total Q10 (ng/mL)
1	1	562	694	48.8	14.2	743
2	1	7700	1000	45.5	22.1	1050
3	1	477	599	35.1	17.0	634
4	1	386	756	52.7	14.4	809
5	1	350	685	43.4	15.8	729
6	2	N.D.	1500	55.3	27.2	1560
7	2	N.D.	950	58.8	16.2	1000
8	2	N.D.	883	23.7	37.3	906
9	2	N.D.	383	26.6	14.4	409
10	2	N.D.	711	33.4	21.3	744

N.D: not detected.

## Data Availability

The original contributions presented in the study are included in the article; further inquiries can be directed to the corresponding author.

## Supplementary Materials

The following supporting information can be downloaded at: https://www.mdpi.com/article/10.3390/metabo16050344/s1, Figure S1: Calibration curves of ubiquinol-10, ubiquinone-10 and Alpha-lipoic acid in spiked pooled serum sample; Table S1: Summary of previously reported LC-MS/MS methods for the quantification of ubiquinol-10, ubiquinone-10 and Alpha-lipoic acid in human biological samples.

## Abbreviations

The following abbreviations are used in this manuscript:

ALA	Alpha-lipoic acid
CE	Collision energy
CoQ_10_	Ubiquinone-10
CoQ_10_H_2_	Ubiquinol-10
CVDs	Cardiovascular diseases
CXP	Collision exit potential
DP	Declustering potential
ECD	Electrochemical detector
EMA	European Medicines Agency
EP	Exit potential
ESI	Electrospray ionization
EtOH	Ethanol
FDA	Food and Drug Administration
H_2_O	Water
HCOOH	Formic acid
HCOONH_4_	Ammonium formate
HPLC	High-performance liquid chromatography
HQC	High-quality control
ICH	International Council for Harmonisation of Technical Requirements for Pharmaceuticals for Human Use
IPA	Isopropanol
IS	Internal standard
LC-MS/MS	Liquid chromatography tandem mass spectrometry
LDL	Low-density lipoprotein
LLE	Liquid–liquid extraction
LOD	Limit of detection
LOQ	Limit of quantitation
LQC	Low-quality control
ME (%)	Matrix effect
MeCN	Acetonitrile
MeOH	Methanol
MQC	Medium-quality control
MRM	Multiple reaction monitoring
QCs	Quality control samples
R^2^	Coefficient of determination
RE (%)	Recovery
ROS	Reactive oxygen species
RP-LC MS/MS	Reverse-phase liquid chromatography tandem mass spectrometry
RSD	Relative standard deviation
t_R_	Retention time
UV	Ultraviolet
XIC	Extracted ion chromatogram
