# Supplementary material for: Integrated Redox Profiling: Simultaneous Determination of Ubiquinol-10, Ubiquinone-10, and Alpha-Lipoic Acid in Serum by LC-MS/MS
Source: Metabolites. 2026 May 20;16(5):344. doi: 10.3390/metabo16050344 (PMC13208553; doi:10.3390/metabo16050344)
Supplement: Supplementary file 1 [file metabolites-16-00344-s001.zip › metabolites-4304763-supplementary.pdf]

Supplementary Information

Table S1. Summary of previously reported LC-MS/MS methods for the quantification of ubiquinol-10, ubiquinone-10 and Alphalipoic acid in human biological samples.

Study	Analytes	Matrix	Sample Preparation	Run Time	LOD/LOQ
Ruiz-Jiménez et al. [13]	Ubiquinol-10<br>Ubiquinone-10	Human serum (100 µL)	PP+LLE+Evaporation	5 min	4.7/15.8<br>1.7/ 4.7
Claessens et al. [11]	Ubiquinol-10<br>Ubiquinone-10	Human plasma (100 µL)	Single step PP	4.2 min	1.0/5.0<br>1.0/10.0
Visconti et al. [31]	Ubiquinone-10	Human plasma (250 µL)	PP+LLE+Evaporation	10 min	5.0/10.0
Chen et al. [33]	Alpha-Lipoic acid	Human plasma (500 µL)	Single step PP	4.5 min	2.0/5.0
**This study**	Ubiquinol-10<br>Ubiquinone-10<br>Alpha-Lipoic acid	Human serum (50 µL)	Unified sample prep with Single step PP	6.5 min	1.2/4.2<br>0.5/1.7<br>0.2/0.7

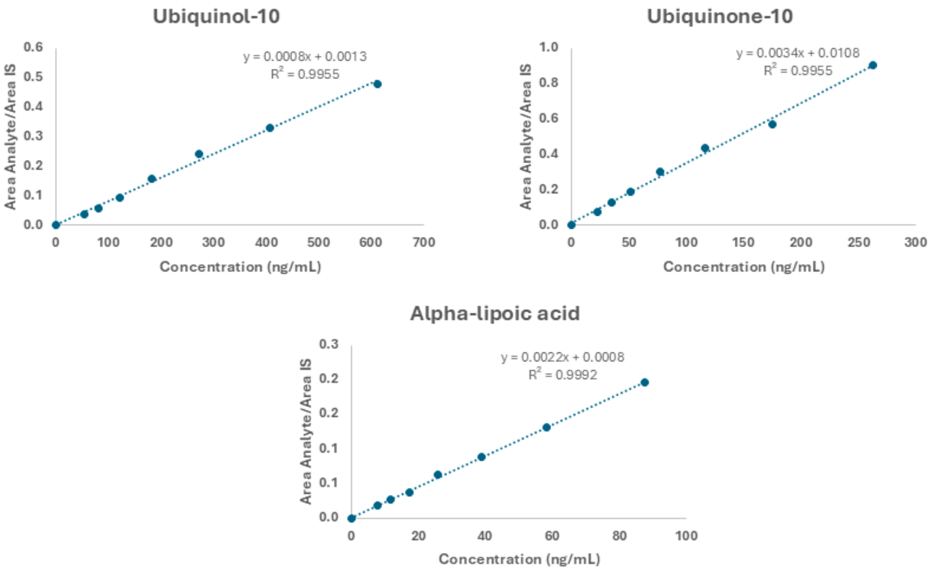

Figure S1. Calibration curves of ubiquinol-10, ubiquinone-10 and Alpha-lipoic acid in spiked pooled serum sample.
